# Particle therapy and nanomedicine: state of art and research perspectives

**DOI:** 10.1186/s12645-017-0029-x

**Published:** 2017-11-21

**Authors:** Sandrine Lacombe, Erika Porcel, Emanuele Scifoni

**Affiliations:** 1 Institut des Sciences Moléculaires d’Orsay (UMR 8214) Bât 351, University Paris Saclay, University of Paris Sud, CNRS, 91405 Orsay Cedex, France; 2 Biophysics Department, GSI Helmholtzzentrum für Schwerionenforschung, 64291 Darmstadt, Germany; 3 TIFPA-INFN, Trento Institute for Fundamental Physics and Applications, University of Trento, 38121 Trento, Italy

**Keywords:** Particle therapy, Proton therapy, Carbon therapy, Radiosensitization, Radio-enhancement, Radioresistance, Nanomedicine, Theranostic, Nanoparticles

## Abstract

Cancer radiation therapy with charged particle beams, called particle therapy, is a new therapeutic treatment presenting major advantages when compared to conventional radiotherapy. Because ions have specific ballistic properties and a higher biological effectiveness, they are superior to x-rays. Numerous medical centres are starting in the world using mostly protons but also carbon ions as medical beams. Several investigations are attempting to reduce the cost/benefit ratio and enlarge the range of therapeutic indications. A major limitation of particle therapy is the presence of low but significant damage induced in healthy tissues located at the entrance of the ion track prior to reaching the tumour. It is thus a major challenge to improve the targeting of the tumours, concentrating radiation effects in the malignance. A novel strategy, based on the addition of nanoparticles targeting the tumour, was suggested over a decade ago to improve the performance of conventional photon therapy. Recently, similar developments have emerged for particle therapy and the amount of research is now exploding. In this paper, we review the experimental results, as well as theoretical and simulation studies that shed light in the promising outcomes of this strategy and in the underpinning mechanisms. Several experiments provide consistent evidence of significant enhancement of ion radiation effects in the presence of nanoparticles. In view of implementing this strategy for cancer treatment, simulation studies have begun to establish the rationale and the specificity of this effect. In addition, these studies will help to outline a list of possible mechanisms and to predict the impact of ion beams and nanoparticle characteristics. Many questions remain unsolved, but the findings of these first studies are encouraging and open new challenges. After summarizing the main results in the field, we propose a roadmap to pursue future research with the aim to strengthen the potential interplay between particle therapy and nanomedicine.

## Introduction

Conventional radiotherapy is applied in 50% of cancer treatments. Based on the properties of high-energy photons to traverse the entire body, this non-invasive method is used to treat deeply seated tumours. However, as the interaction of photons is not tissue specific, severe side effects or even secondary cancers may be induced when healthy tissues are damaged. It is thus a major challenge to develop new strategies and improve the tumour selectivity of radiation effects.

The enrichment of tumours with high-Z compounds has been proposed as a new strategy to improve the effects of radiation as due to the amplification of primary (electronic) processes. To avoid confusion with radiosensitizing drugs, those compounds that make cells more sensitive to radiation, such as DNA repair inhibitors, oxygen transporters [see for instance (Lawrence et al. 2003)], in this review, we use the term “nano-radio-enhancers” (NRE) to distinguish these compounds.

The principle of radio-enhancement was first demonstrated using metallic complexes to increase the effects of high-energy photons [see (Kobayashi et al. 2010) for a review]. The clinical use of these compounds is, however, limited by the lack of tumour selectivity. Hence, nanoparticles (NPs) have been proposed as a more efficient means to improve the concentration of active products in the tumour and, as a consequence, to improve the tumour targeting of radiation effects. The selective delivery of NPs is due to the enhanced permeability and retention effect (EPR) when the systems are small enough (diameter <200 nm) to permeate through the tumour blood vessel walls (Jäger et al. 2013). Tumour targeting may also be achieved when nanoparticles are functionalized with tumour specific agents such as antibodies or other peptides [see (Friedman et al. 2013) for review]. Thus, the combination of radiation therapies with nanomedicine opens a new range of treatments (Kong et al. 2008). Hainfeld et al. (2008) were the first to show that 1.9 nm gold core NPs prolong the life of mice treated with 160 kV X-rays. Gold NPs are presently the most well studied agents [see (Her et al. 2017) and (Haume et al. 2016) for review]. Other sophisticated NPs, composed of other heavy elements such as hafnium (Maggiorella et al. 2012) and gadolinium (Sancey et al. 2014) developed by Nanobiotix (Paris, France) and NH TherAguix (Villeurbanne, France) respectively, are already being transferred to the clinic.

**Fig. 1:**
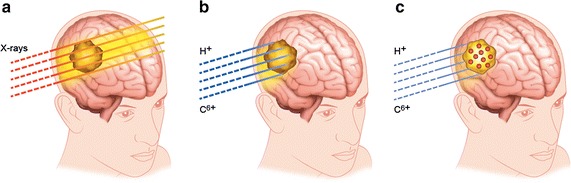
Illustration of **a** highly penetrating X-ray radiation propagation leading to damage in healthy tissues, **b** ballistic effects of ions with negligible radiation effects after the tumour but still significant effects at the entrance of the track, and **c** improvement of ion radiation effects in the tumour in the presence of nanoparticles, which opens the possibility to reduce the dose to the patient and the dose deposition in the tissues located prior to reaching the tumour

Although conventional radiotherapy has been tremendously improved (e.g., with the IMRT technique), the use of highly penetrating photons remains critical for the treatment of tumours located in close vicinity of sensitive organs (i.e. eyes, brain, neck) and the treatment of paediatric cases, where damage of surrounding tissues can have severe consequences. The latter are mainly related to the geometry of the irradiation (e.g. in a typical craniospinal irradiation for a medulloblastoma, the dose to the spine is extremely dangerous) and to the young age of the patients, which emphasizes later risk effects (Armstrong et al. 2010). Moreover, conventional radiotherapy is not able to eradicate rare but highly aggressive radioresistant cancers such as glioblastoma and chordoma, for which the treatment outcomes remain poor. For these cases, treatment by high-energy ions such as protons (proton therapy) and carbon ions (carbon therapy) is being proposed as an alternative (Durante et al. 2017). The main advantage of ion beams (70–400 MeV/amu) stems from their property to penetrate tissues over several centimeters and deposit the maximum energy at the end of their track, where the ionization cross section of the medium is extremely large and at a depth dependent from their initial energy, forming the so called Bragg peak in a depth dose profile (Schardt et al. 2010). Thus, the beam may be tuned by modulating its energy to target the tumour without damaging the tissues located at a deeper position [see Fig. 1)]. Moreover, thanks to a larger relative biological effectiveness (RBE) associated to ion beam radiation as compared to X-rays due to its more densely ionizing feature providing greater cell killing for the same amount of delivered dose (Scifoni 2015), particle therapy is also the most efficient method to treat radioresistant tumours (Ares et al. 2009; Schlaff et al. 2014; Kamada et al. 2015; Durante et al. 2017). Carbon ions in particular can, in some cases, be four times more efficient than X-rays (Loeffler and Durante 2013; Kamada et al. 2015). Particle therapy is thus considered, at least for a number of indications, superior to conventional radiotherapy (Baumann et al. 2016) and, in spite of the high costs, new centres of proton therapy and carbon therapy are developing worldwide. In fact, beyond the 74 centres already in operation as of April 2017, 83 new centres have already started construction [e.g. in Dallas (USA) and Lanzhou (China)] and at least another 40 (e.g. in Australia, India, Denmark and Netherlands) are in the planning stages [see (Jermann 2015; Zietman 2016) for recent printed reviews and the PTCOG dedicated website for most updated data: https://www.ptcog.ch/index.php/facilities-in-operation].

Particle therapy is delivered with two different modalities. One is the passively modulated broad beam modality, which consists of a beam shaped to the target with a spread out Bragg peak (SOBP). The second is the recent pencil beam active scanning mode, where a beamlet of a few mm is scanned, spot by spot, on the tumour, modulating the energy for each depth slice (Schardt et al. 2010). Because of its larger degradation of the beam through the beamline materials, the broad beam modality usually provides a larger entrance channel dose, as compared to the pencil beam (Shiomi et al. 2016).

Hence, because of the physical profile of the beam, a low but significant dose deposited by the ions in the tissues located before reaching the tumour [see Fig. 1b] is unavoidable. Moreover, damage to surrounding tissues may be caused by motion and a range of other uncertainties.

To overcome these limitations, the addition of NREs to the tumour is proposed as a challenging strategy to amplify the effect of ion radiation locally and thus reduce the total dose to the patient. The use of contrast agents, in particular, offers the possibility to follow the biodistribution of the agent as well as to image the tumour just prior to or during the treatment. While nanomedicine is now approaching a clinical stage in conventional radiotherapy, only few studies have been dedicated to the combination of high-Z NREs with ion beam modalities.

This review summarizes the first experimental and modelling studies that display and tentatively describe the effects of different radio-enhancers, including metallic complexes and NPs, used to improve the performance of particle beam treatments, e.g. protons, helium and carbon ion radiation. The first section exposes the major results reported on the effects of (i) platinum complexes activated by different ion radiations (helium, carbon, iron), (ii) gold NPs combined with proton radiation and (iii) platinum NPs and gadolinium-based nanoagents (AGuiX) combined with carbon radiation. In the second section, the recent modelling and simulation studies dedicated to radio-enhancement induced by ion radiation are collected together with a summary of the known results and the remaining open questions to be faced.

## Overview of experimental studies

### Combination of platinum complexes with various ion radiations

The proof of principle of this strategy was first demonstrated with platinum complexes (chloroterpyridine platinum, PtTC) used as radio-enhancers (presented below). Given that nanosize bio-damage is the most lethal for living cells, the amplification of these types of damage is a major challenge of the strategy. Hence, DNA plasmids have been used as nano-bioprobes to detect and quantify the induction of nanosize bio-damage. The study of Usami et al. (2005) demonstrated for the first time that the presence of platinum based complexes strongly amplifies the induction of these types of damage when helium ions (143 MeV/amu initial energy and Linear Energy Transfer (LET) of 2.24 keV μm^−1^) are used as ionizing radiation (Usami et al. 2005). It was demonstrated that this amplification of the ion radiation effects is mediated, for >90%, by the production of reactive oxygen species (ROS) (indirect effects). Thus, the amplification of ion radiation by high-Z agents was explained by (i) the activation of the high-Z atoms by incident ions or electrons of the track due to Coulombic interaction, (ii) de-excitation and electron emission and (iii) production of radicals in the medium.

Later, the same group observed that the effects of medical carbon ions (276 MeV/amu, 13 keV/μm and 109 MeV/amu, 25.6 keV/μm) and iron ions (400 MeV/amu, 200 keV/μm) may also be used to improve treatment (Usami et al. 2007). Here again, the important role of water radicals was demonstrated. Interestingly, the radio-enhancement effect was found to be lower with high LET iron ions. This was attributed to a decrease of the indirect effect due to an overproduction of hydroxyl radicals that recombine and produce peroxide (Hirayama et al. 2009).

**Fig. 2:**
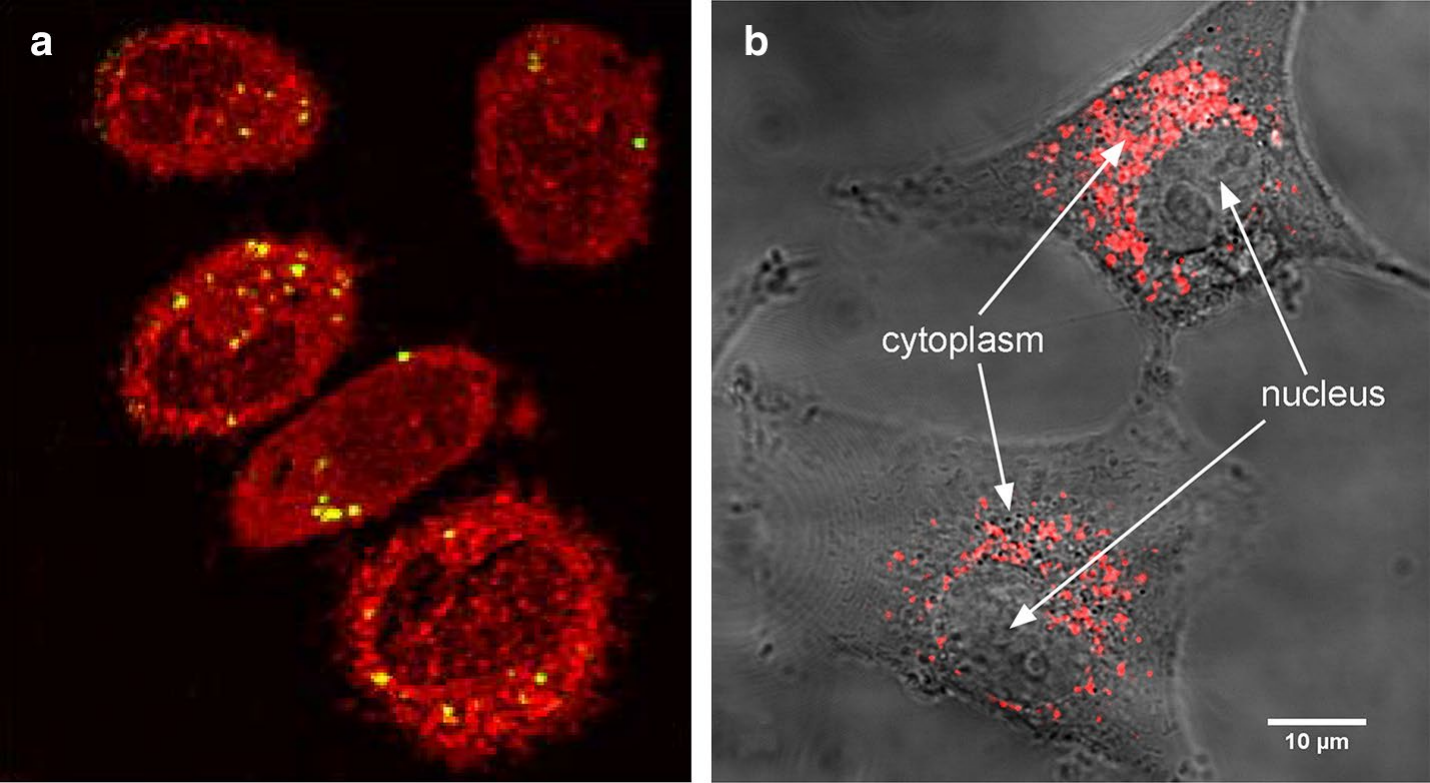
**a** Localization of platinum complexes (yellow) in the cytoplasm of the cells (red). The darker areas correspond to the cell nucleus. Adapted from (Usami et al. 2008a); **b** Localization of gadolinium-based nanoparticles (red) in the cytoplasm of glioblastoma cells. Adapted from (Stefančíková et al. 2014)

**Fig. 3:**
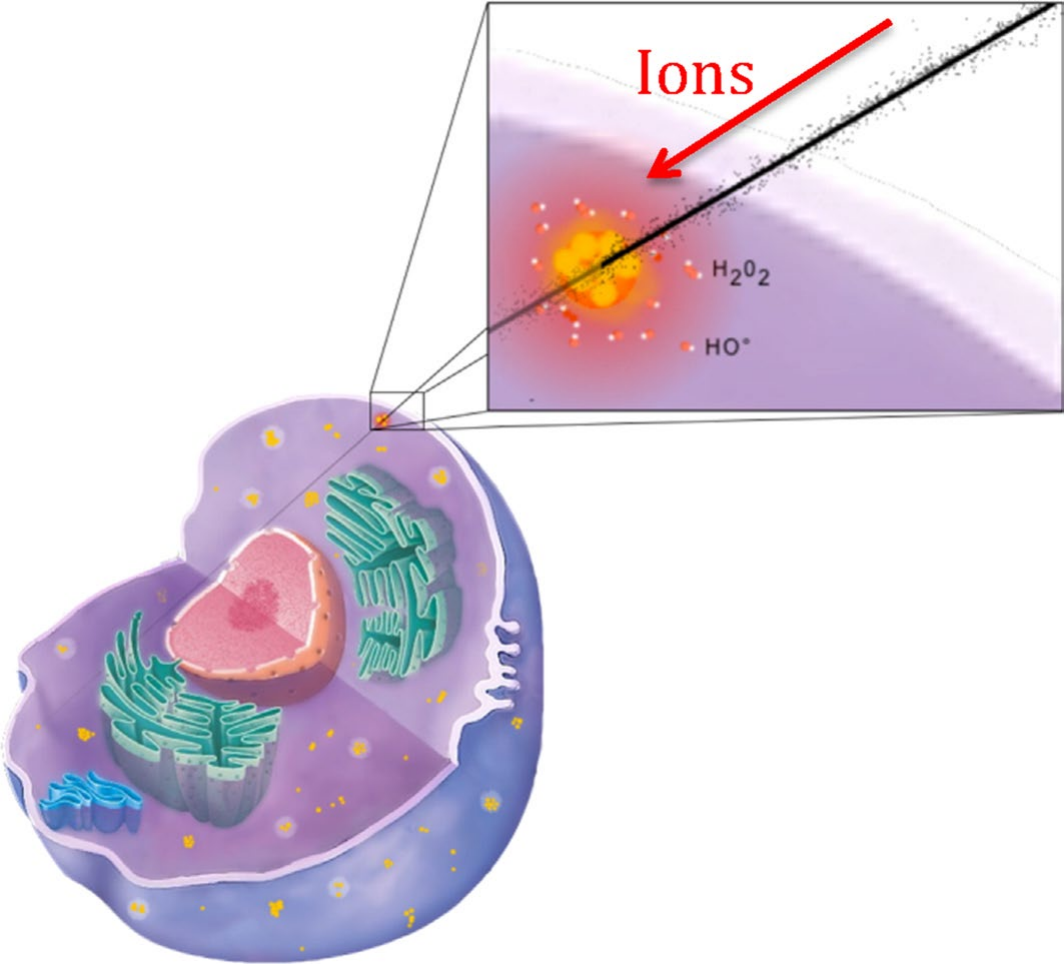
Sketch of nanoscale impact initiated by nanoparticles in the cytoplasm (Adapted from (Porcel et al. 2014))

These molecular scale experiments were followed by cellular scale proof of principle studies. The effect of the efficacy of the same platinum complexes (chloroterpyridine platinum) to amplify the effects of carbon ions was shown in vitro (Usami et al. 2008a). This study confirmed that hydroxyl radicals play a major role. Interestingly, it was found that the enhancement efficacy per track is larger at the track end (high LET), while from simple mechanistic arguments one would expect the contrary, i.e. a larger relative effect for a more photon-like (low-LET) condition (see the next section for details). More importantly, microscopy measurements demonstrated, for the first time, that cell killing is enhanced despite the localization of the radio-enhancing agents in the cytoplasm, and not in the nucleus, of the cells (see Fig. 2a). This was a major outcome, which already showed that radio-enhancement by high-Z agents activated by ionizing radiation begins in the cell cytoplasm (see Fig. 3 for a possible model).

These studies opened the perspectives to improve the performance of particle therapy using high-Z complexes. They shed light on putative early stage mechanisms involved in the enhancement of radiation effects, and on the role of hydroxyl radicals in particular. Unfortunately, these complexes, which are not tumour specific and not detectable by medical imaging (CT and MRI), are not suitable for clinical transfer.

As an alternative, nanotechnologies open new perspectives to target tumours. The effect of nanoparticles, combined with particle radiation, has been probed with high-energy protons and medical carbon ions (see below).

### Combination of nanoparticles with proton radiation

The effectiveness of high-Z nanoparticles to improve the performance of proton radiation was first demonstrated by Kim et al. (2010). They observed that small nanoparticles (diameter 1.9–14 nm), composed of gold or iron, enhance the regression of CT26 mouse tumours treated by fast protons (45 MeV-beam, pristine Bragg Peak, in the entrance, LET not specified). They also observed, with in vitro experiments, that cell killing is enhanced when CT 26 cells are loaded with nanoparticles. Thus, the group demonstrated that in vivo impact is strongly correlated with increasing cell killing. This shows the impact of cellular scale effects on the body scale impact. The mechanism proposed by the authors has proven to be controversial. It was argued that proton induced X-ray emission (PIXE) cannot account as the major process in the amplification of radiation effects (Dollinger 2011). Indeed, the probability for the nanoparticles to be activated by the X-rays induced by PIXE was proved to be very low, as explained in detail by Dollinger (2011).

The efficiency of gold to enhance the effects of proton radiation was confirmed in vitro by Polf et al. (2011). This group observed a significant increase (15–19% RBE at 10 and 50% survival, respectively) of prostate tumour cell mortality when loaded with gold containing phage-nanoscaffolds (44 nm diameter, 1 ng gold per cell) and irradiated by 160 MeV protons, with cells located in a large (10 cm) SOBP at a dose averaged LET of approximately 12 keV/μm. Kim et al. (2012) later confirmed that the amplification of tumour regression and mice survival treated by 40 MeV protons (complete tumour regression >37% with 100–300 mg gold/kg) is related to ROS production in tumour cells (Kim et al. 2012). This finding is in full agreement with the conclusion of the above-mentioned studies using platinum complexes.

Jeynes et al. (2014) found that 50 nm citrate capped gold nanoparticles do not amplify the effects of 3 MeV protons on RT112 bladder cancer cells (Jeynes et al. 2014). However, Li et al. (2016) observed, using epidermoid carcinoma cells (A 431), that 2 MeV protons have greater effects when the cells are loaded with 5 or 10 nm PEG amine coated gold nanoparticles (Li et al. 2016). Surprisingly, the nanoparticles were found located in the nucleus, unlike most other studies using gold nanoparticles [see (Moser et al. 2016)]. They highlighted the important role of hydroxyl radicals. Interestingly, the effect of NPs increased with the beam LET (amplifying factors: 25–40% with 10 and 25 keV/μm LETs beams, respectively).

Recent molecular scale experiments performed with platinum and gadolinium nanoparticles, activated by 150 MeV protons, highlighted the amplification of nanosize bio-damage (Schlathölter et al. 2016). Here again, the role of hydroxyl radicals was shown. More importantly, the radio-enhancement effect was found to be greater at the end of the ion track.

In summary, these studies reinforce the perspective of using NREs to concentrate the effects of proton radiation at the track end in the tumours.

### Combination of nanoparticles with carbon ions

The group of Lacombe (Porcel et al. 2010) was the first to demonstrate the efficacy of small (3 nm) metallic nanoparticles to amplify the effects of medical carbon beams (provided by HIMAC, the hadrontherapy center of Chiba, Japan). This was performed at a molecular scale using platinum nanoparticles (coated with polyacrylic acid, PAA) activated by 290 MeV/amu carbon ions at two LETs (13 and 110 keV/μm) (Porcel et al. 2010). Here again, the role of ROS in the amplification of nanosize bio-damage was highlighted. As mentioned in more detail in the next section on the mechanistic analysis, nanoparticles may be activated by charged particles (incident ions or secondary electrons of the track) by Coulombic interaction (including ionization and surface plasmon excitation channels). Radicals are produced due to the interaction of electrons emitted by the nanoparticles, but also by the capture of electrons from surrounding water molecules. Interestingly, a significant role of the nanoparticle structure was observed, and metallic nanoparticles were found to be more efficient than metallic complexes at the same concentration. This was attributed to the size of the volume perturbed by the radio-enhancers which, in the case of nanoparticles, is of the order of a few nanometers. The emission of electrons and consecutive ROS clusters produced in this nano-volume can favour the induction of complex damage. In contrast, molecular agents amplify the electron emission in smaller volumes, which is less efficient to induce molecular damage of nanometer size. Hence, nanoparticles do not merely increase the number of breaks but rather improve the quality of the radiation effect.

The biological response to this early stage nanoscale perturbation may be diverse and is the subject of several cell studies.

Kaur et al. (2013) observed amplification of carbon ion radiation in tumour cells (HeLa) loaded with gold nanoparticles (Kaur et al. 2013). A dose enhancement factor (DEF) close to 40% RBE was obtained using 62 MeV carbon ion beam irradiation, 290 keV/μm LET. This should be compared to the effects obtained when nanoparticles are activated by 1 MeV gamma radiation. The authors obtained a higher effect than the one observed with the proton beam irradiation observed by Polf et al. (2011). However, since the groups used different cell models, cell uptake and cell sensitivity may well play an important role.

The amplification of medical carbon radiation effects was then evidenced with gadolinium-based nanoagents (AGuiX from Nano-H, Lyon, France). These theranostic agent have unique multimodal properties, including improvement of MRI contrast and enhancement of radiation effects (Porcel et al. 2014). This study demonstrated that cell killing induced by carbon ion radiation (290 MeV/amu at SOBP beam) is augmented even with a low concentration of gadolinium. The relationships between cellular and molecular impacts and the role of ROS were also shown. Noticeably, the gadolinium-based nanoparticles were found located in the cytoplasm [see Fig. 2b (Stefančíková et al. 2014)], which confirms that enhancement of cell killing is initiated in the cytoplasm (likely via the production of radical clusters). This study opened the first opportunity to introduce theranostic in carbon therapy.

More recently, the enhancement of cell killing of HeLa cells loaded with 14 nm gold nanoparticles and irradiated by carbon ions, has been reported (Liu et al. 2015). They established that the enhancement does not increase with the concentration of nanoparticles, which indicates that this effect is not related to the physical dose. This confirms the conclusion of Porcel et al. (2014) and Mc Mahon et al. (2011) who stipulate that the effect of nanoparticles is due to the confinement in nanometer size volumes of the electronic perturbation and ROS production, which increases the toxicity of radiation. Here again, the nanoparticles were found located in the cytoplasm.

An exhaustive summary of the experimental studies reported in this first part is presented in Table 1.

**Table 1:** Chronological overview of experimental studies on radio-enhancement of fast ion radiation effects by high-Z compounds

Ion type	E	LET (keV/μm)	Dose rate (Gy/min)	Sample	Radio-enhancer	Data	Evidence of radical effects	Localisation	Refs./year
Helium	143 MeV/amu	2.24 (HIMAC)	4	pBr322 plasmid	PtTC complex	SSB/DSB	Yes^a^	–	Usami et al. (2005)
Carbon	276 MeV/amu 109 MeV/amu	13.4 (HIMAC) 25.6	7 6.2	pBr322 plasmid	PtTC complex	SSB/DSB	Yes	–	Usami et al. (2007)
Iron	400 MeV/amu 100 MeV/amu	200 (HIMAC) 550	–	pBr322 plasmid	PtTC complex	SSB/DSB	Yes	–	
Carbon	290 MeV/amu	13 (HIMAC) 70	7 6.2	CHO-K-1 cells	PtTC complex	SF	Yes	Yes	Usami et al. (2008a)
Helium	150 MeV/amu	2 & 7 (HIMAC)	4	CHO-K-1 cells	PtTC complex	SF	Yes	Yes	
Carbon	276 MeV/amu	13.4 (HIMAC)	4	pBr322 plasmid	PtNP-PAA 3 nm		Yes	–	Porcel et al. (2010)
Carbon	290 MeV/amu	13.2 (HIMAC) 110	7	pBr322 plasmid	PtNP-PAA 3 nm		Yes	–	
Protons	40 MeV	1 (Pristine peak) 35 (Bragg Peak)	480	CT26 cells	AuNPs 2–13 nm	SF	–	–	Kim et al. (2010)
Protons	40 MeV	1 (Pristine peak) 35 (Bragg Peak)	480	CT26 cells	FeNPs 14 nm	SF	–	–	
Protons	40 MeV	1 (Pristine peak) 35 (Bragg peak)	–	CT26 T in mice	AuNPs 2–13 nm	T growth/mice survival	–	–	
Protons	80 MeV	–	4	NSA fibrocarcoma in mice	Cis-Pt	T growth/mice survival	–	Yes	Terakawa et al. (2011)
Protons	160 MeV	SOBP	–	DU 145 Prostate carcinoma cells	Phage plus AuNPs 44 nm	SF	–	–	Polf et al. (2011)
Helium	150 MeV/amu	2.3 (HIMAC)	4	pBr322 plasmid	PtNP-PAA 3 nm	SSB/DSB	Yes	–	Porcel et al. (2012)
Carbon	290 MeV/amu	13.2 110 (HIMAC)	7	pBr322 plasmid	PtNP-PAA 3 nm	SSB/DSB	Yes	–	
Protons	45 MeV	Pristine Bragg Peak Entrance plateau	0.5–0.7	CT26 T in mice	AuNPs 14 nm	T uptake/T growth/Mice survival	Yes	–	Kim et al. (2012)
Protons	45 MeV	Pristine Bragg Peak Entrance plateau	0.5–0.7	CT26 T in mice	FeNPs 10.6 nm	T uptake/T growth/Mice survival	Yes	–	
Carbon	62 MeV	290		HeLa	Glu-AuNPs 6 nm		–	–	Kaur et al. (2013)
Carbon	270 MeV/amu	13 (HIMAC)	7	pBr322 plasmid	GdBNP 3 nm	SSB/DSB	Yes	–	Porcel et al. (2014)
Helium	150 MeV/amu	2.3 (HIMAC)	4	pBr322 plasmid	GdBNP 3 nm	SSB/DSB	–	–	
Carbon	270 MeV/amu	13	7	CHO	GdBNP 3 nm	SF		Yes	
Protons	3 MeV	12	–	RT112	Citrate-AuNPs 50 nm		Yes	Yes	Jeynes et al. (2014)
Protons	2 MeV	10 and 25	1	A431	Amine PEG capped AuNPs 5 & 10 nm	SF	Yes	Yes	(Li et al. (2016)
Carbon	165 MeV/amu	70 (HIRFL)	0.4	HeLa	AuNPs 14 nm (1.5–15 μg/mL)	Viability, SF, cell cycle	Yes	Yes	Liu et al. (2015, 2016)
Protons	150 MeV	0.44 3.6	–	pBr322 plasmid	PtNP-PAA 3 nm	SSB/DSB	Yes	–	Schlathölter et al. (2016)
Protons	150 MeV	0.44 3.6	–	pBr322 plasmid	GdBNP 3 nm	SSB/DSB	Yes	–	

SSB/DSB stay for single/double strand breaks, while SF is survival fraction

Studies at molecular scale (black), in vitro (blue) and in vivo (red) levels are reported. The method of LET measurements are not specified in the papers, but are usually determined on the basis of measurements performed with ionization chambers in water

^a^Yes means availability of data concerning the radical production and the NP localisation in cells. The absence of ticks is indicating the absence of data

## Simulation studies

Modelling of the nanoscopic mechanisms involved in nanoparticle induced radio-enhancement was first undertaken in the case of photon irradiation. The amplification of radiation effects in this case was explained in terms of a nanoscale enhancement of the local dose in close vicinity to the NPs. This was demonstrated by McMahon et al. (2011) and recently refined by Brown and Currell (2017), thus explaining the results of several experiments through adapting the Local Effect Model (LEM) (Scholz and Kraft 1996) initially developed for ion beams. This model, in its simpler formulation (LEM I), predicts a higher cell killing for higher densely ionizing (LET) radiation, correlating a higher spatial concentration of ionizations on a biological target, and then the induction of more severe damage to a higher probability to induce a lethal effect and than cell death. It was seen that simply including the high local enhancement of the dose due to Auger electrons can lead to a significant effect on the radial dose, which then induces an increase in cell killing quantified by a Sensitizing Enhancement Ratio (SER), i.e. a ratio of doses giving the same biological effect with and without sensitizer, in a way similar to an RBE (McMahon et al. 2011).

**Fig. 4:**
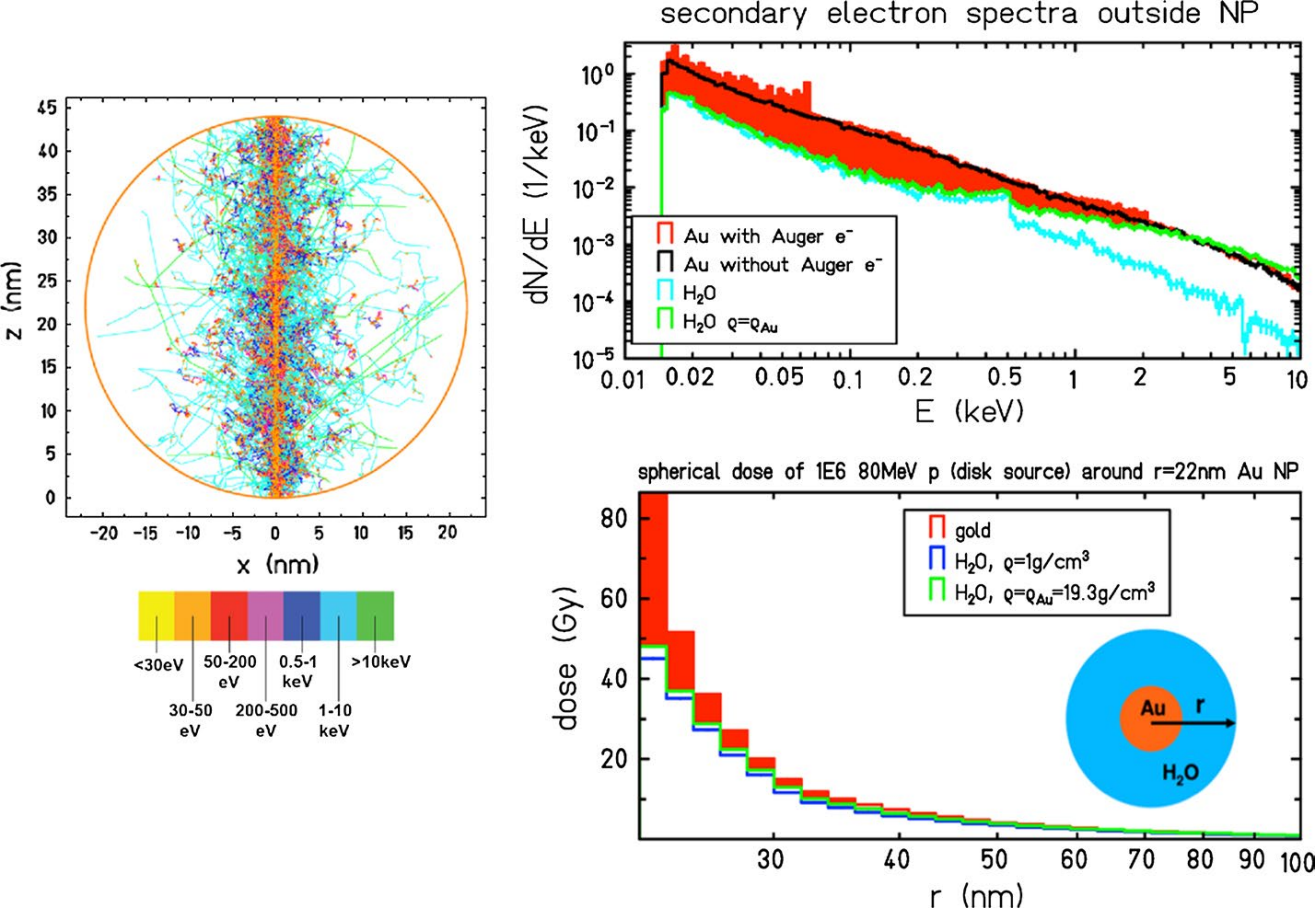
Model analysis of NP sensitization with proton irradiation, according to (Wälzlein et al. 2014). Left: Simulated track of a 80 MeV proton across a gold NP with 2 nm radius, including all secondary electrons, performed with TRAX. Right-upper: spectra of electrons escaping the NP, as compared to the case when the NP is replaced by normal water or water with a density (*ρ*) equal to gold. Right-lower: corresponding dose enhancement (see text for details) (Adapted from (Wälzlein et al. 2014))

In the case of ion beam irradiation, an enhancement of radiation effects was observed in the presence of nanoparticles either at the molecular (DNA damage), in vitro (cell killing) and in vivo (mouse tumour regression) levels, as discussed in the previous section and listed in Table 1. However, the mechanistic explanation of local dose enhancement provided for photons is not the same as for ions. In the studies with photons, it was shown that a large increase in the radial dose profile was induced in the presence of NP as compared to photon irradiation in water, enough to justify the sizeable difference in the yield of severe damage. However, in the case of ions, the dose is already highly localized along the tracks, and an extremely high local dose would be required to induce an additional impact on the damage concentration, without even accounting for over-kill effects. In this case, the enhancement of radiation effects is not, as yet, fully understood. The first study approaching this problem (Wälzlein et al. 2014) was conducted using the particle track structure code TRAX (Krämer and Kraft 1994) to analyse, at a nanoscale level, a possible dose enhancement in high-Z nanoparticles (Au, Pt, Ag, Fe and Gd) traversed by proton beam (see Fig. 4). It was found that a relevant increase in local dose around the nanoparticle could be computed, but the relative enhancement was much smaller than that observed in photon irradiation. Moreover, the simulation was performed in the condition of ion traversing across the nanoparticle, which with typical fluences adopted in proton therapy (10^6^ to 10^9^ cm^−2^) is very rare. Thus, the dose enhancement effect occurring in the case of an ion traversal should be weighted by this very low probability to occur (≈10^−3^ to 10^−4^). In total, this would lead to a noticeably reduced overall dose enhancement effect. This study has shown a larger effect of gold and platinum, as compared to other high-Z materials, in acting as dose enhancers. More importantly, it demonstrated that, for proton radiation, a significant dose enhancement effect can be observed, mostly due to Auger electrons and consecutive cascades. However, this process is not sufficient to justify any overall macroscopic effect such as those observed in several experiments.

The amplification effect of ion radiation by high-Z NPs may be explained by other mechanisms, such as modification of the radiation chemistry pathways and enhancement of radical mediated component of radiation damage, as suggested with X-rays (Sicard-Roselli et al. 2014).

Gao and Zheng (2014) explored different proton energies and found that a larger number of electrons escape the nanoparticles for lower primary ion energy. These electrons have lower energies and shorter ranges compared to those induced by more energetic protons (Gao and Zheng 2014). Lin et al. (2014) attempted to establish comparative figures of merit between protons and different types of photon radiation (Lin et al. 2014) and proposed a model for biological effect calculation (Lin et al. 2015) based on the Local Effect Model. The result pointed out the need of a much higher nanoparticle uptake in the case of protons as compared to photons, in order to observe a similar enhancement effect. This concentration should be even higher for protons of lower energies for the emitted electrons of lower range to reach and affect sensitive cell components.

Verkhovtsev et al. (2015a, b) proposed the idea of a new  channel through surface plasmon excitation, which was shown to strongly link to a large production of secondary electrons, thus arguing a new pathway for dose enhancement [Verkhovtsev et al. (2015a, b]. The authors showed, for 1 MeV protons, an increase of an order of magnitude in the emitted electron spectra, as compared to direct ionization.

Other studies, using Monte Carlo calculations, have been performed focusing on macroscopic dose enhancement due to the absorbed physical dose only (Ahmad et al. 2016; Cho et al. 2016). The effect was found to be very small for realistic values of NP concentrations.

A recent study (Martínez-Rovira and Prezado 2015) confirmed that a nanoscale dose enhancement, based on physical boost of electron production alone, cannot explain the amplification effect observed in experiments and that radiation chemistry or biological pathways should also be taken into account (Wälzlein et al. 2014). A critical summary of Monte Carlo studies on proton interaction with NP has been collected in Verkhovtsev et al. (2017).

A recent study attempted to include the physico-chemical and chemical stage in this process for protons of 2 to 170 MeV traversing a gold NP, using a combination of GEANT4 and GEANT4-DNA (Tran et al. 2016). Despite the underestimation of secondary electrons production at low energy inherent to the model, this study emphasized an interesting “radiolysis enhancement factor “, i.e., an increased radical production due the presence of the gold NP, which increases with the energy of the incident particle.

**Fig. 5:**
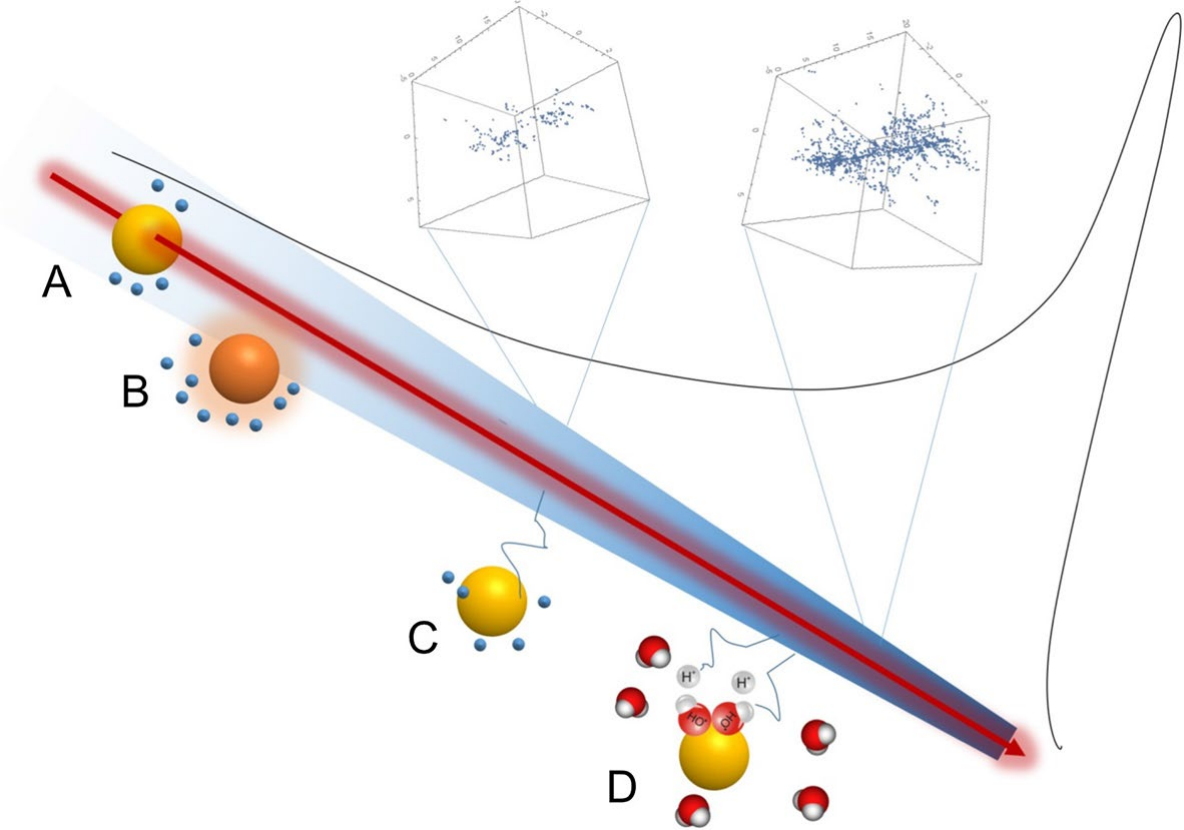
Sketch of possible mechanisms involved in the enhancement of ion beam effects by radio-enhancing NP. A) Direct traversal: enhanced electron production from Auger electrons and Auger cascades. B) Plasmon excitation from a close distance and following coupling with strong electron production. C) Electrons produced in the primary track impinging the NP, which produces additional electron emission. D) Enhancement of radiolytic species due to a catalytic effect of the NPs, promoting the dissociation of excited water molecules, amplifying radical production. These mechanisms may take place anywhere along the track. Insets: Monte Carlo simulations of secondary electron tracks in a 20 nm segment at different depths of the ion track

In Fig. 5, we show a scheme that summarizes all the mechanisms proposed in these studies.

Thus, despite the fact that several questions have been answered, modelling of the enhancement of ion beam effects with NPs is just at its initial stage. There is a large need for further studies. In particular, before entering the radiobiological effects, the first parameters to be verified are the cross sections of the pure physical processes, which are needed in the simulation codes. While many studies are focused on detecting a biological effect, the physics itself has still to be fully elucidated. For example, both elastic and inelastic cross sections in high-Z materials like gold have still not been characterized in detail, and relevant differences appear, e.g. when using the standard Livermore library (Wälzlein et al. 2014). Studies in this direction are now ongoing, providing, for the moment, a partial confirmation of the validity of the cross section sets used in TRAX (Hespeels et al. 2017).

As for the search of the ideal conditions of radio-enhancement, only effects of incident protons have been simulated, and there is no indication of a possible trend of the track structure effect, thus emphasizing an ion type dependence (beyond pure LET), as has been demonstrated for the RBE (Friedrich et al. 2013). As for the pure energy (or LET) dependence, despite some indications, there is still not a complete explanation of the enhancement effect. In particular, from experiments, this dependence appears counter-intuitive, pointing to a larger effect for higher LET, while one should expect a larger enhancement for a more “photon-like” radiation type. The challenges arising from these studies will probably stimulate research not only to shed light on the specific mechanism, but also on reconsidering the general paradigm of radiation bio-damage (Scifoni 2015).

In addition, the role of oxygenation of the medium (quantified by the Oxygen Enhancement Ratio—OER) may be significant. The OER with ion beams shows a strong peculiarity, decreasing with high LET (Furusawa et al. 2000). Thus far, the OER effect associated with the presence of nanoparticles has not yet been considered, aside from a study with photons where anoxic cells appeared to be not sensitized by NPs (Jain et al. 2014). However, this effect could be different with ion beams, and the potential to additionally sensitize hypoxic cells with NPs is very attractive. Last, but not least, it will be necessary to explicitly study the case of radio-enhancement mediated by NPs in the cytoplasm. In fact, as discussed above, it is now almost established, from most of the prior studies, that the enhancement of cell killing is induced by nanosensitisers located in the cytoplasm (Usami et al. 2008b; Porcel et al. 2010; Stefančíková et al. 2014), despite the fact that, as mentioned in the previous section, a few studies have also found NPs in the nucleus (Li et al. 2016). This type of study was initiated for photons, pointing to mitochondria as possible sensitive targets (McMahon et al. 2017). In the case of ions, these targets will have a completely different and probably more complex scenarios.

## Conclusions and outlook

The development of nanoagents to improve the performance of particle therapy is only at its beginning. Several studies already demonstrated the feasibility of this strategy, but the efficacy of nanoparticles must be further optimized to be of clinical interest for radio-oncologists.

The results obtained with several nanoparticles are already promising but greater efforts are needed to improve active tumour targeting, renal clearance, and detection of the agents by medical imaging (CT or MRI). The nanoagents of the future will have various designs (i.e. nanoparticles, nanocages, nanocarriers [see for instance (Horcajada et al. 2010; Yu et al. 2012; Kunz-Schughart et al. 2017)] and will offer unique perspectives to combine different modalities using the same compound. For instance, NPs able to act on the immune system, such as those proposed for some cancer treatments (Dimitriou et al. 2017; Ebner et al. 2017), will be of particular interest for particle therapy.

In parallel, the mechanistic sequences involved in the enhancement of ion radiation effect, which is needed for predictive assessments, are not yet fully revealed, but a number of clear pictures are emerging. However, in order to appropriately simulate the enhancement effect and introduce the concept in treatment planning, the explicit description of the radiation chemistry, initiated after the physical step, will be required.

The association of particle therapy and nanomedicine is a new era. Its evolution depends on the capacity of the different communities to share their expertise in developing competitive nanoagents and predictive models. In this context, a collaborative European research programme entitled Marie Curie ITN “ARGENT” (http://itn-argent.eu) has been initiated (Bolsa Ferruz et al. 2017).

## Abbreviations

CEA: Atomic Energy Center
CHO: Chinese Hamster Ovary
CPBM: Centre de Photonique Bio-Medical
CTCF: corrected total cell fluorescence
DMEM: Dulbecco’s Modified Eagle Medium
EL4: mouse lymphoma cell line
GBM: glioblastoma multiforme
GdBN: gadolinium-based nanoparticles
GdBN-Cy5.5: GdBN labelled with cyanine 5.5
HBSS: Hank’s Balanced Salt Solution
EELS: electron energy loss spectroscopy
EF: enhancing factor
FITC: Fluorescein IsoThioCyanate
ICP: inductively coupled plasma
LET: linear energy transfer
NPs: nanoparticles
SD: standard deviation
SF: surviving fraction
SR-DUV: synchrotron-radiation deep UV
SQ20B: human head and neck squamous cells carcinoma cell line
TEM: transmission electron microscopy
U87: human glioblastoma cell line

## References

[CR1] Ahmad R, Royle G, Lourenco A (2016). Investigation into the effects of high-Z nano materials in proton therapy. Phys Med Biol.

[CR2] Ares C, Hug EB, Lomax AJ (2009). Effectiveness and safety of spot scanning proton radiation therapy for chordomas and chondrosarcomas of the skull base: first long-term report. Int J Radiat Oncol Biol Phys.

[CR3] Armstrong GT, Stovall M, Robison LL (2010). Long-term effects of radiation exposure among adult survivors of childhood cancer: results from the childhood cancer survivor study. Radiat Res.

[CR4] Baumann M, Krause M, Overgaard J (2016). Radiation oncology in the era of precision medicine. Nat Rev Cancer.

[CR5] Bolsa Ferruz M, Ivošev V, Haume K (2017). New research in ionizing radiation and nanoparticles: the ARGENT project. Nanoscale Insights Ion-Beam Cancer Ther.

[CR6] Brown JMC, Currell FJ (2017). A local effect model-based interpolation framework for experimental nanoparticle radiosensitisation data. Cancer Nanotechnol.

[CR7] Cho J, Gonzalez-Lepera C, Manohar N (2016). Quantitative investigation of physical factors contributing to gold nanoparticle-mediated proton dose enhancement. Phys Med Biol.

[CR8] Dimitriou NM, Tsekenis G, Balanikas EC (2017). Gold nanoparticles, radiations and the immune system: current insights into the physical mechanisms and the biological interactions of this new alliance towards cancer therapy. Pharmacol Ther.

[CR9] Dollinger G (2011). Therapeutic application of metallic nanoparticles combined with particle-induced x-ray emission effect. Nanotechnology.

[CR10] Durante M, Orecchia R, Loeffler JS (2017). Charged-particle therapy in cancer: clinical uses and future perspectives. Nat Rev Clin Oncol.

[CR11] Ebner DK, Tinganelli W, Helm A (2017). The immunoregulatory potential of particle radiation in cancer therapy. Front Immunol.

[CR12] Friedman AD, Claypool SE, Liu R (2013). The smart targeting of nanoparticles. Curr Pharm Des.

[CR13] Friedrich T, Durante M, Scholz M (2013). Particle species dependence of cell survival RBE: evident and not negligible. Acta Oncol.

[CR14] Furusawa Y, Fukutsu K, Aoki M (2000). Inactivation of aerobic and hypoxic cells from three different cell lines by accelerated (3)He-, (12)C- and (20)Ne-ion beams. Radiat Res.

[CR15] Gao J, Zheng Y (2014). Monte Carlo study of secondary electron production from gold nanoparticle in proton beam irradiation. Int J Cancer Ther Oncol.

[CR16] Hainfeld JF, Dilmanian FA, Slatkin DN, Smilowitz HM (2008). Radiotherapy enhancement with gold nanoparticles. J Pharm Pharmacol.

[CR17] Haume K, Rosa S, Grellet S (2016). Gold nanoparticles for cancer radiotherapy: a review. Cancer Nanotechnol.

[CR18] Her S, Jaffray DA, Allen C (2017). Gold nanoparticles for applications in cancer radiotherapy: mechanisms and recent advancements. Adv Drug Deliv Rev.

[CR19] Hespeels F, Heuskin AC, Scifoni E (2017). Backscattered electron emission after proton impact on carbon and gold films: experiments and simulations. Nucl Instruments Methods Phys Res Sect B Beam Interact with Mater Atoms.

[CR20] Hirayama R, Matsumoto Y, Kase Y (2009). Radioprotection by DMSO in nitrogen-saturated mammalian cells exposed to helium ion beams. Radiat Phys Chem.

[CR21] Horcajada P, Chalati T, Serre C (2010). Porous metal–organic-framework nanoscale carriers as a potential platform for drug delivery and imaging. Nat Mater.

[CR22] Jäger E, Jäger A, Chytil P (2013). Combination chemotherapy using core-shell nanoparticles through the self-assembly of HPMA-based copolymers and degradable polyester. J Control Release.

[CR23] Jain S, Coulter JA, Butterworth KT (2014). Gold nanoparticle cellular uptake, toxicity and radiosensitisation in hypoxic conditions. Radiother Oncol.

[CR24] Jermann M (2015). Particle therapy statistics in 2014. Int J Part Ther.

[CR25] Jeynes JCG, Merchant MJ, Spindler A (2014). Investigation of gold nanoparticle radiosensitization mechanisms using a free radical scavenger and protons of different energies. Phys Med Biol.

[CR26] Kamada T, Tsujii H, Blakely EA (2015). Carbon ion radiotherapy in Japan: an assessment of 20 years of clinical experience. Lancet Oncol.

[CR27] Kaur H, Pujari G, Semwal MK (2013). In vitro studies on radiosensitization effect of glucose capped gold nanoparticles in photon and ion irradiation of HeLa cells. Nucl Instrum Methods Phys Res Sect B Beam Interact with Mater Atoms.

[CR28] Kim J-K, Seo S-J, Kim H-T (2012). Enhanced proton treatment in mouse tumors through proton irradiated nanoradiator effects on metallic nanoparticles. Phys Med Biol.

[CR29] Kim J-K, Seo S-J, Kim K-RK-H (2010). Therapeutic application of metallic nanoparticles combined with particle-induced x-ray emission effect. Nanotechnology.

[CR30] Kobayashi K, Usami N, Porcel E (2010). Enhancement of radiation effect by heavy elements. Mutat Res.

[CR31] Kong T, Zeng J, Wang X (2008). Enhancement of radiation cytotoxicity in breast-cancer cells by localized attachment of gold nanoparticles. Small.

[CR32] Krämer M, Kraft G (1994). Calculations of heavy-ion track structure. Radiat Environ Bioph.

[CR33] Kunz-Schughart LA, Dubrovska A, Peitzsch C (2017). Nanoparticles for radiooncology: mission, vision, challenges. Biomaterials.

[CR34] Lawrence TS, Blackstock AW, McGinn C (2003). The mechanism of action of radiosensitization of conventional chemotherapeutic agents. Semin Radiat Oncol.

[CR35] Li S, Penninckx S, Karmani L (2016). LET-dependent radiosensitization effects of gold nanoparticles for proton irradiation. Nanotechnology.

[CR36] Lin Y, McMahon SJ, Paganetti H, Schuemann J (2015). Biological modeling of gold nanoparticle enhanced radiotherapy for proton therapy. Phys Med Biol.

[CR37] Lin Y, McMahon SJ, Scarpelli M (2014). Comparing gold nano-particle enhanced radiotherapy with protons, megavoltage photons and kilovoltage photons: a Monte Carlo simulation. Phys Med Biol.

[CR38] Liu Y, Liu X, Jin X (2015). The dependence of radiation enhancement effect on the concentration of gold nanoparticles exposed to low- and high-LET radiations. Phys Medica.

[CR39] Liu Y, Liu X, Jin X (2016). The radiation enhancement of 15 nm citrate-capped gold nanoparticles exposed to 70 keV/m carbon ions. J Nanosci Nanotechnol.

[CR40] Loeffler JS, Durante M (2013). Charged particle therapy—optimization, challenges and future directions. Nat Rev Clin Oncol.

[CR41] Maggiorella L, Barouch G, Devaux C (2012). Nanoscale radiotherapy with hafnium oxide nanoparticles. Futur Oncol.

[CR42] Martínez-Rovira I, Prezado Y (2015). Evaluation of the local dose enhancement in the combination of proton therapy and nanoparticles. Med Phys.

[CR43] McMahon SJ, Hyland WB, Muir MF (2011). Biological consequences of nanoscale energy deposition near irradiated heavy atom nanoparticles. Sci Rep.

[CR44] McMahon SJ, McNamara AL, Schuemann J (2017). Mitochondria as a target for radiosensitisation by gold nanoparticles. J Phys: Conf Ser.

[CR45] Moser F, Hildenbrand G, Müller P (2016). Cellular uptake of gold nanoparticles and their behavior as labels for localization microscopy. Biophys J.

[CR46] Polf JC, Bronk LF, Driessen WHP (2011). Enhanced relative biological effectiveness of proton radiotherapy in tumor cells with internalized gold nanoparticles. Appl Phys Lett.

[CR47] Porcel E, Li S, Usami N (2012). Nano-sensitization under gamma rays and fast ion radiation. J Phys: Conf Ser.

[CR48] Porcel E, Liehn S, Remita H (2010). Platinum nanoparticles: a promising material for future cancer therapy?. Nanotechnology.

[CR49] Porcel E, Tillement O, Lux F (2014). Gadolinium-based nanoparticles to improve the hadrontherapy performances. Nanomed Nanotechnol Biol Med.

[CR50] Sancey L, Lux F, Kotb S (2014). The use of theranostic gadolinium-based nanoprobes to improve radiotherapy efficacy. Br J Radiol.

[CR51] Schardt D, Elsässer T, Schulz-Ertner D (2010). Heavy-ion tumor therapy: physical and radiobiological benefits. Rev Mod Phys.

[CR52] Schlaff CD, Krauze A, Belard A (2014). Bringing the heavy: carbon ion therapy in the radiobiological and clinical context. Radiat Oncol.

[CR53] Schlathölter T, Lacombe S, Eustache P (2016). Improving proton therapy by metal-containing nanoparticles: nanoscale insights. Int J Nanomed.

[CR54] Scholz M, Kraft G (1996). Track structure and the calculation of biological effects of heavy charged particles. Adv Sp Res.

[CR55] Scifoni E (2015). Radiation biophysical aspects of charged particles: from the nanoscale to therapy. Mod Phys Lett A.

[CR56] Shiomi M, Mori S, Shinoto M (2016). Comparison of carbon-ion passive and scanning irradiation for pancreatic cancer. Radiother Oncol.

[CR57] Sicard-Roselli C, Brun E, Gilles M (2014). A new mechanism for hydroxyl radical production in irradiated nanoparticle solutions. Small.

[CR58] Stefančíková L, Porcel E, Eustache P (2014). Cell localisation of gadolinium-based nanoparticles and related radiosensitising efficacy in glioblastoma cells. Cancer Nanotechnol.

[CR59] Terakawa A, Ishii K, Yamazaki H (2011). PIXE analysis of a murine solid tumor treated with proton therapy combined with cisplatin. X-Ray Spectrom.

[CR60] Tran HN, Karamitros M, Ivanchenko VN (2016). Geant4 Monte Carlo simulation of absorbed dose and radiolysis yields enhancement from a gold nanoparticle under MeV proton irradiation. Nucl Instrum Methods Phys Res Sect B Beam Interact with Mater Atoms.

[CR61] Usami N, Furusawa Y, Kobayashi K (2005). Fast He^2+^ ion irradiation of DNA loaded with platinum-containing molecules. Int J Radiat Biol.

[CR62] Usami N, Furusawa Y, Kobayashi K (2008). Mammalian cells loaded with platinum-containing molecules are sensitized to fast atomic ions. Int J Radiat Biol.

[CR63] Usami N, Furusawa Y, Kobayashi K (2008). Mammalian cells loaded with platinum-containing molecules are sensitized to fast atomic ions. Int J Radiat Biol.

[CR64] Usami N, Kobayashi K, Furusawa Y (2007). Irradiation of DNA loaded with platinum containing molecules by fast atomic ions C(6 +) and Fe(26 +). Int J Radiat Biol.

[CR65] Verkhovtsev A, Korol AV, Solov’yov AV (2017). Irradiation-induced processes with atomic clusters and nanoparticles. Nanoscale insights into ion-beam cancer therapy.

[CR66] Verkhovtsev AV, Korol AV, Solov’yov AV (2015). Revealing the mechanism of the low-energy electron yield enhancement from sensitizing nanoparticles. Phys Rev Lett.

[CR67] Verkhovtsev AV, Korol AV, Solov’yov AV (2015). Electron production by sensitizing gold nanoparticles irradiated by fast ions. J Phys Chem C.

[CR68] Wälzlein C, Scifoni E, Krämer M, Durante M (2014). Simulations of dose enhancement for heavy atom nanoparticles irradiated by protons. Phys Med Biol.

[CR69] Yu MK, Park J, Jon S (2012). Targeting strategies for multifunctional nanoparticles in cancer imaging and therapy. Theranostics.

[CR70] Zietman AL (2016). Particle therapy at the “Tipping Point”: an introduction to the red journal’s special edition. Int J Radiat Oncol.

